# Two Patients With Brain Tumors Who Received Bevacizumab and Radiotherapy: Optic Neuropathy and Quality-of-Life Issues

**DOI:** 10.6004/jadpro.2013.4.4.5

**Published:** 2013-07-01

**Authors:** D. Beverly Fu, Daniela Alexandru, Dana M. Curticiu, Yao Fu*, Daniela A. Bota

**Affiliations:** From University of California at Irvine, Irvine, California; *School of Nursing, Harbin Medical University, China

**ABSTRACT**

**Case Studies**

**CASE 1**

The first case we present is that of Mr. R., a 70-year-old Hispanic man with a diagnosis of unresectable World Health Organization grade II oligodendroglioma in bilateral frontal lobes. Although he did not have a high-grade malignancy, he received the same therapy as would a patient with a high-grade tumor. Mr. R. underwent standard radiotherapy and temozolomide (TMZ) chemotherapy. Bevacizumab (Avastin) was initiated at the time of tumor progression, which was 2 months after the completion of radiotherapy. At that time, Mr. R.’s visual acuity was 20/25 in both eyes without any corrective lenses. Pseudoprogression was ruled out with magnetic resonance (MR) spectroscopy and brain positron emission tomography (PET)/computed tomography (CT) before the start of bevacizumab treatment.

Mr. R. developed optic neuropathy 7 months after the initiation of bevacizumab therapy. His MRI remained stable throughout the bevacizumab therapy, with no tumor progression on either T1 contrast-enhanced or T2/FLAIR sequences. He lost the vision in his left eye first, followed by the vision in his right eye 2 months later. Neuro-ophthalmologic evaluation revealed no pallor in either optic nerve. The mean doses of radiation used were 50.6 Gy to the right optic nerve, 37.9 Gy to the left optic nerve, and 52.8 Gy to the optic chiasm. The tumor received 59.4 Gy in 33 fractions from both initial and intensity-modulated radiation therapy (IMRT) boost radiation treatments (see planning maps in Figures 1 and 2). Mr. R. developed bevacizumab-induced hypertension and was on amlodipine 5 mg daily. He had no evidence of tumor involvement with the optic nerves on MRI and no preexisting or new-onset diabetes mellitus.

**Figure 1:**
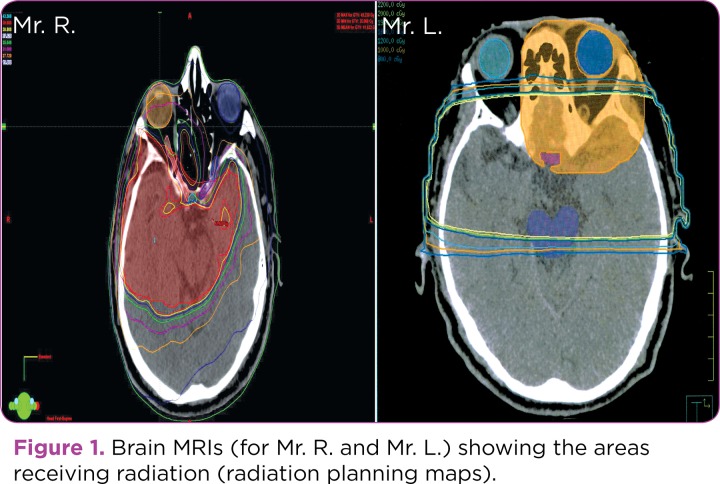
Figure 1. Brain MRIs (for Mr. R. and Mr. L.) showing the areas receiving radiation (radiation planning maps).

**Figure 2:**
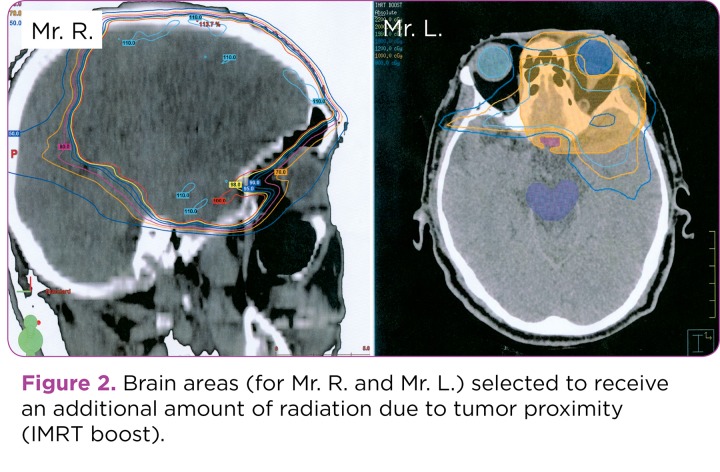
Figure 2. Brain areas (for Mr. R. and Mr. L.) selected to receive an additional amount of radiation due to tumor proximity (IMRT boost).

After the onset of his blindness, Mr. R. developed depression, severe anxiety, and hallucinations related to having difficulty identifying the times of day. He was not on any medication that could have contributed to his anxiety or depression, such as a steroid or levetiracetam. Visual seizures were ruled out at the time. Mr. R. was also seeing vivid pictures. After a formal psychiatric evaluation, he was diagnosed with Charles Bonnet syndrome. Additionally, Mr. R. suffered several falls at home due to the loss of his vision. Mrs. R., the patient’s wife and primary caregiver, also developed anxiety as a result of taking care of her husband. Mr. R. eventually succumbed to his disease.

**CASE 2**

The second case is that of Mr. L., an 82-year-old Asian man with glioblastoma multiforme in the left frontal lobe. Mr. L. received concomitant TMZ chemotherapy and radiotherapy. His tumor recurred 2 months after the completion of radiotherapy, and he was started on bevacizumab therapy. At that time, Mr. L.’s visual acuity was assessed as normal with corrective lenses to 20/40 in both eyes.

Mr. L. developed optic nerve neuropathy 14 months after the initiation of bevacizumab. He lost the vision in his right eye first, followed by that in his left eye 2 to 3 months later. Mr. L. met neither the McDonald nor the Revised Assessment in Neuro-Oncology (RANO) criteria for tumor progression. Neuro-ophthalmologic evaluation revealed right optic nerve pallor and atrophy and left optic nerve pallor. Mr. L.’s eyes were positive for retinal pigment epithelium changes as well as sclerotic vessels.

Mr. L. received 28.67 Gy to the right optic nerve, 32.7 Gy to the left optic nerve, and 46.49 Gy to the optic chiasm. The tumor received a total radiation dose of 60 Gy from both initial and IMRT boost treatments in 30 fractions (see Figures 1 and 2).

Mr. L. had preexisting mild hypertension and was on irbesartan 150 mg daily. He had no preexisting or new-onset diabetes mellitus and no evidence of malignancy involvement in his optic nerves upon MRI. After losing his vision, Mr. L. had a flat affect and decreased interest in performing the activities of daily living. He developed depression but refused to take antidepressants. He suffered several falls in the home. Mr. L.’s son and grandson became actively involved in his care, which increased the family’s stress level. Mr. L. continued receiving bevacizumab therapy for a total of 2 years following his disease recurrence. Despite prolonged local disease control, Mr. L.’s quality of life decreased dramatically after he lost his ability to see.

High-grade malignant gliomas, also known as glioblastoma multiforme (GBM) and anaplastic astrocytomas, are the most common primary brain tumors. These tumors are associated with a devastating prognosis. The current standard therapy for these malignancies is concomitant radiation therapy and temozolomide (TMZ) chemotherapy (Sherman et al., 2009). Bevacizumab (Avastin) is a monoclonal antibody against vascular endothelial growth factor (VEGF) that inhibits angiogenesis by preventing VEGF receptor activation (Khasraw, Simeonovic, & Grommes, 2012). This molecule showed promising results in phase II clinical trials (Nagane et al., 2012; Lai et al., 2008) and is currently used as either monotherapy or adjuvant therapy with irinotecan after the first recurrence of GBM following treatment with radiotherapy and TMZ chemotherapy (Khasraw, Simeonovic, & Grommes, 2012).

Recently, a phase II clinical pilot study regarding the use of bevacizumab in combination with TMZ and regional radiation therapy for up-front treatment of patients with newly diagnosed GBM showed promising results (Lai et al., 2008).

However, as this article was going to press, results of the Radiation Therapy Oncology Group 0825 phase III study were presented at the plenary session of the 2013 annual meeting of the American Society of Clinical Oncology (Gilbert et al, 2013). The data showed that although adding bevacizumab to the standard of care (chemoradiation with temozolomide) for first-line treatment of patients with GBM was associated with longer progression-free survival, one of the study’s endpoints, the combination did not reach the predetermined significance criterion. No improved was reported for overall survival, the study’s other endpoint.

## Optic Neuropathy

Optic neuropathy is a rare but well-documented complication of radiation treatment for brain tumors that is associated with severe consequences for affected patients (Chamberlain, Raizer, Schiff, & Sherman, 2010; Lessell, 2004; Sherman et al., 2009). The relationship between optic neuropathy and bevacizumab treatment is controversial at this point and requires further studies (Nagane et al., 2012).

Radiation-induced optic neuropathy (RION) has been described previously. Mihalcea and Arnold have indicated that RION can be acute or delayed: It can occur during radiation therapy or anytime from 3 months to 10 years later (Mihalcea & Arnold, 2008). Serova et al. reported RION in six patients with brain tumors who were administered radiotherapy and radiosurgery within standard therapeutic doses (Serova, Eliseeva, Lazareva, Arutiunov, & Shishkina, 2001). In one case, a patient with a tumor close to the left cavernous sinus developed RION 4 years after receiving treatment to a total dose of 60 Gy (Serova et al., 2001). Subsequently, magnetic resonance imaging (MRI) showed an enlarged, enhancing optic chiasm in the absence of tumor recurrence (Serova et al., 2001).

Although both Mr. R. and Mr. L. were elderly men, age does not seem to be a factor in the development of RION. According to a review of the literature, the age range for RION diagnosis is 32 to 68 years old. Hyperbaric oxygen therapy has been used to treat optic neuropathy. However, it is controversial and has been associated with very limited efficacy in preserving visual function (Levy & Miller, 2006). Finger (2007) reported that intravitreal bevacizumab treatment improved vision and recommended further investigation (Sherman et al., 2009; Desai, Pratt, Lentzner, & Robinson, 2001).

We propose that when used 2 to 3 months after radiation therapy, bevacizumab may hinder the healing process of the optic nerves and may increase the risk of RION and blindness in patients with GBM. In this article we have presented two cases of RION in patients undergoing treatment with bevacizumab for GBM. Both Mr. R. and Mr. L. had baseline neuro-ophthalmologic exams for comparison and received standard radiation to their brain tumors (60 Gy in 30 daily fractions). As both patients had frontal lobe tumors, they presented with frontal lobe syndrome that manifested in personality changes and impaired thinking, but their symptoms varied based on the extent of damage. Evans, Fletcher, and Wormald (2007) advised that patients with visual impairment have a higher incidence of depression as opposed to anxiety. We have found that vision loss exacerbates patients’ depression and apathetic symptoms. As a result, the patient’s quality of life deteriorates abruptly, and these new symptoms add to significant caregiver stress.

## Discussion

As mentioned previously, bevacizumab is an antiangiogenic agent that is widely used today in the treatment of malignant gliomas. The major side effects of this therapy are hemorrhage, hypertension, thromboembolism, proteinuria, gastrointestinal perforation, impaired wound healing, and sensory neuropathy. Less than 2% of patients may experience blurred vision (Lai et al., 2008; Nagane et al., 2012; Sherman et al., 2009).

In 2009, Sherman et al. reported the first series of six patients from five institutions that developed RION after being treated with radiotherapy followed by bevacizumab for their GBM (Sherman et al., 2009). In this study, the patients had tumors located in the frontal and temporal lobes. The mean dose of radiation to the optic chiasm was between 3,504 and 6,218 cGy. The radiation dose to the left optic nerve was from 3,073 to 4,302.4 cGy, and the radiation dose to the right optic nerve was from 2,250 to 5,309 cGy (Sherman et al., 2009). The mean target volume was from 56.6 to 66 Gy (Sherman et al., 2009). The investigators concluded that the six patients’ optic neuropathy was most likely related to the arterial thrombosis and neovascularization postradiation with delayed ischemia after treatment with bevacizumab (Sherman et al., 2009).

Our patients, Mr. R. and Mr. L., are quite similar to the patients in the series presented by Sherman and colleagues (Table 1). Both patients developed severe optic neuropathy after the start of their bevacizumab treatment, and they both received TMZ chemotherapy and standard-dose radiotherapy. The diagnosis of optic neuropathy was confirmed by a comprehensive neuro-ophthalmologic evaluation.

**Table 1:**
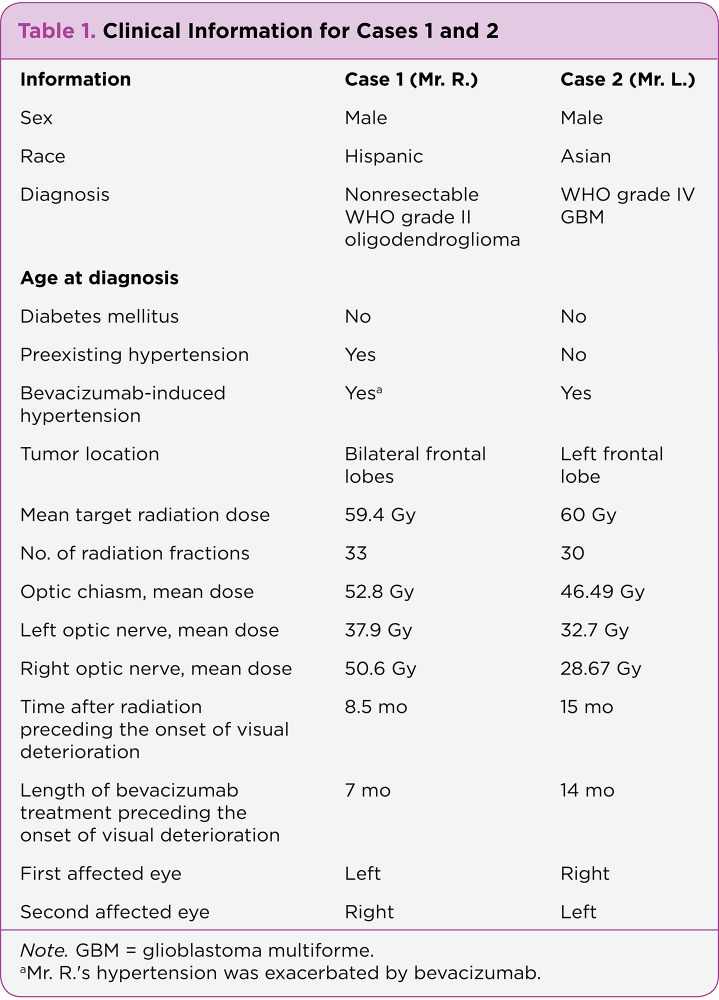
Table 1. Clinical Information for Cases 1 and 2

The implications of the diagnosis of optic neuropathy are far-reaching. As described previously, Mr. R. and Mr. L. developed depression and psychiatric disturbances (Table 2). They lost interest in performing the activities of daily living. In addition, the families of the patients were under increased stress, as the level of care required by a blind patient with GBM is much more intensive and involved. Desai et al. explored the quality-of-life issues of patients with visual loss by examining 2,530 subjects randomly; they determined that vision loss adversely impacts patients’ safety, independence in daily activities, emotional contentment, and use of community resources (Desai et al., 2001). The investigators stated that older adults with vision loss have a high risk of falling, and the risk for mild or moderate depression is 57.2% (Desai et al., 2001).

**Table 1:**
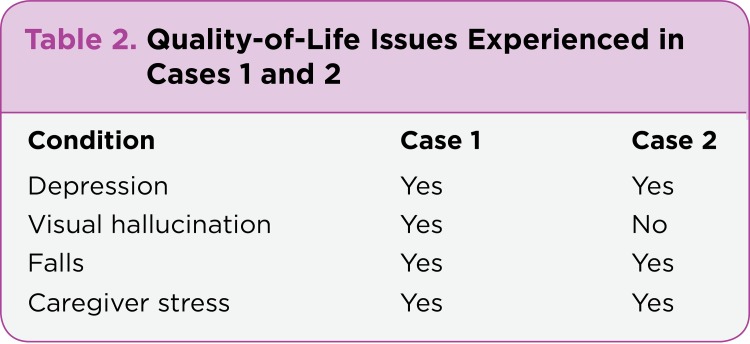
Table 1. Quality-of-Life Issues Experienced in Cases 1 and 2

## Detection and Management

Vision loss in the GBM patient can lead to devastating effects on quality of life. As such, it is crucial that oncology advanced practitioners be alert to the possibility of RION. Early identification and management of RION can be extremely beneficial for these patients. For those patients with frontal tumors receiving radiation followed by bevacizumab, close follow-up with the neuro-ophthalmology service is recommended. In addition to routine eye examination, we recommend psychiatric evaluation and home health safety evaluations to improve the quality of care and to help prevent functional decline in these patients at risk of developing RION. It is also important to have a multidisciplinary team in place, including a clinical social worker, to provide support for the patient’s family.

## Conclusions

In summary, we believe that bevacizumab administration increases the risk of RION if administered 2 to 3 months after the end of radiation treatment. Close monitoring and follow-up of the visual apparatus, as well as psychiatric evaluation and assessment of home health safety, are essential in preventing further neurologic decline and decrease in quality of life.

## Acknowledgments

The authors would like to acknowledge support from the National Cancer Institute, UCI Cancer Center Award No. P30CA062203.
